# Use of a Pedicled Anterolateral Thigh Flap as Fasciocutaneous and Chimeric With Vastus Lateralis Muscle for Bilateral Trochanteric Radionecrosis Reconstruction: A Case Report

**DOI:** 10.7759/cureus.78188

**Published:** 2025-01-29

**Authors:** Georgios E Papanikolaou, Marios Papadakis, Ilias Iliadis, Dimitrios N Varvarousis, Efstathios G Lykoudis

**Affiliations:** 1 Department of Plastic Surgery, Metropolitan General Hospital, Athens, GRC; 2 School of Medicine, University of Ioannina, Ioannina, GRC; 3 Department of Surgery, University of Witten Herdecke, Wuppertal, DEU; 4 Department of Plastic Surgery, University Hospital of Ioannina, Ioannina, GRC; 5 Department of Orthopaedics, University Hospital of Ioannina, Ioannina, GRC

**Keywords:** anterolateral thigh flap, chimeric anterolateral thigh flap, radiation-induced skin ulcers, soft tissue reconstruction, trochanteric radionecrosis

## Abstract

Pelvic radiation for gynecological malignancies may induce several acute and late complications. Among those, skin and soft tissue radionecrosis at the trochanteric region represents a rare event, associated with significant morbidity. The therapeutic approach for those deep and large trochanteric defects is challenging, requiring a combination of radical debridement of the necrotic tissue and reconstruction using pedicled or free flaps. Accordingly, we present our experience with the use of a pedicled anterolateral thigh flap as fasciocutaneous and chimeric with the vastus lateralis muscle for the reconstruction of a late bilateral trochanteric skin and soft tissue radionecrosis. The wounds were completely healed without evidence of recurrence at the 24-month postoperative follow-up. We emphasize the importance of using well-vascularized and bulky flaps for successful filling and coverage of large post-radiation trochanteric defects to achieve good functional and aesthetic outcomes and to prevent recurrence.

## Introduction

Radiation therapy has been widely used throughout the years for the effective treatment of different types of malignancies, with considerable improvement in survival rates. Particularly, radiotherapy is used in gynecologic cancers to reduce recurrence and therefore patients’ morbidity and mortality rates. However, the effects of radiation on normal tissues can be devastating with a significantly negative impact on patients’ quality of life. Those effects can be classified as acute when observed within six months from the irradiation and late as they can appear after six months or years from the exposure to irradiation [1]. It is estimated that about 3% to 5% of the patients who underwent radiotherapy will experience delayed radiation-related complications such as soft tissue and bone radionecrosis, which is defined as tissue necrosis due to capillary damage, tissue hypoxia, hypocellularity, and fibrosis of the irradiated tissue bed [2].

Since there are no effective strategies for the prevention of radiation-induced tissue necrosis, radiotherapy for pelvic malignancies such as gynecologic cancers can lead to severe late complications, including skin and soft tissue ulceration resulting in large tissue defects [3]. Patients who receive pelvic radiation for gynecologic malignancies may experience the late effects of the radiation toxicity due to the anatomic locations and the concomitant chemotherapy and/or surgery, mainly involving the gastrointestinal (enteritis, proctitis, fistula, obstruction) and genitourinary (cystitis, fistula, strictures, vaginal stenosis) systems, as well as the skin (ulceration, necrosis, infection) and the bones (osteopenia, osteoradionecrosis) [3]. The therapeutic approach of those radionecrotic lesions can be challenging, requiring careful preoperative physical and imaging examination as well as the selection of the appropriate reconstructive method. The goal is the complete healing of the radionecrotic area without recurrence.

Accordingly, we present a rare case of bilateral trochanteric (relating to the upper part of the thigh at the great trochanteric region) radionecrosis that occurred 30 years after radiotherapy for uterine cancer, which was successfully treated with a pedicled fasciocutaneous anterolateral thigh (ALT) flap on the right side and a pedicled chimeric ALT-vastus lateralis muscle (ALT-VL) flap on the left side. The pedicled ALT flap is a surgical technique that uses tissue from the anterolateral part of the thigh with its blood supply intact. We emphasize the importance of combining muscle and fasciocutaneous flaps to provide adequate skin and soft tissue padding for the coverage of similar large tissue defects. Additionally, we highlight the need for long-term vigilance, since delayed effects of radiotherapy may occur many years later. Moreover, given the limited scientific data, our surgical approach aimed to contribute to the literature for the development of effective approaches for similar radiation-related complications. 

## Case presentation

An 80-year-old Caucasian female patient presented with bilateral trochanteric skin and soft tissue radionecrosis after radiotherapy for uterine cancer over a 30-year period. She complained of chronic and persistent pain associated with light limitation of the hip movements for more than a year, while she reported the presence of a low amount of clear drainage at the left trochanteric area lasting approximately six months. An additional aggravating factor was the chronic irritation of the trochanteric region from shear injury and repeated pressure associated with her advanced age. Previous treatments included only the administration of antibiotics and analgesics. Medical history included arterial hypertension, atrial fibrillation, and hyperlipidemia, without other relevant comorbidities. Physical examination revealed the presence of skin hyperpigmentation and atrophy associated with tissue retraction, while at the left trochanteric area, a fistula was evident. Textural changes included xerosis, while subcutaneous fibrosis was evident. Consequently, the patient underwent magnetic resonance imaging (MRI) of the pelvis and hips, which confirmed the clinically suspected bilateral soft tissue trochanteric radionecrosis and the presence of a fistula formation between the skin and the trochanteric bursa on the left side, without involvement of the hip joint. Wound cultures from the fistula were negative for microbial colonization, while blood inflammatory markers were within the normal range (white blood cell level at 8.5 x 103/μL, hemoglobin level at 11.5 g/dL, erythrocyte sedimentation rate at 15 mm/h, and C-reactive protein at 3.5 mg/L). Consequently, we excluded the presence of inflammation and the treatment of the affected areas in multiple stages.

**Figure 1:**
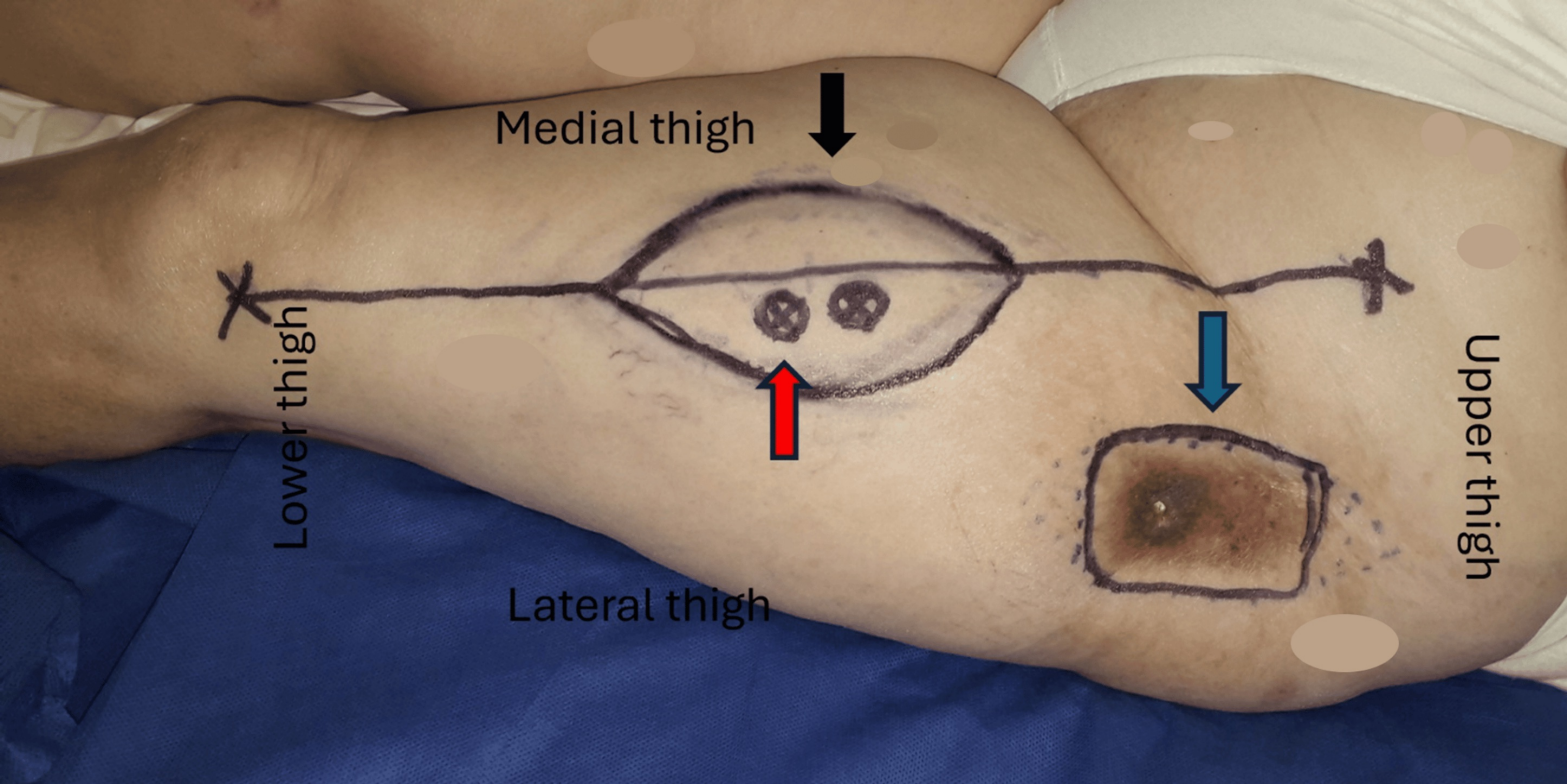
Preoperative markings at the left side showing the anterolateral thigh flap (black arrow), the detection of the most distal perforator (red arrow), and the area of radionecrosis at the trochanteric region (blue arrow). The flap size was approximately 13 cm x 9 cm and was centered over the selected perforators, while the marked size of the defect was 10 cm x 8 cm.

**Figure 2:**
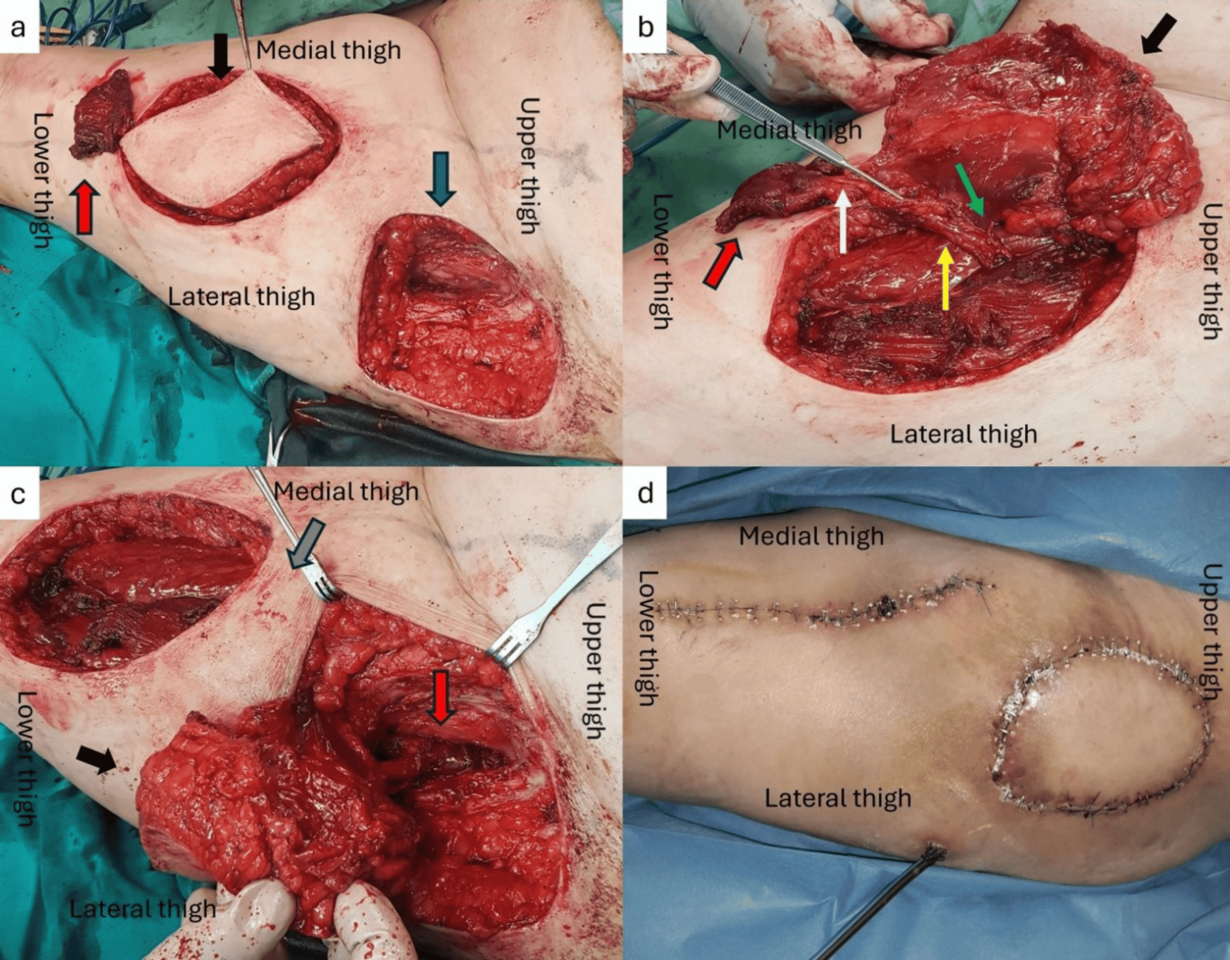
Intraoperative details of the reconstruction of the left trochanteric area (a) Harvesting of the chimeric anterolateral thigh (ALT) (black arrow) and vastus lateralis muscle (VL) (red arrow) flap, and the resulting defect at the trochanteric area after radical debridement (blue arrow); (b) Dissection of the chimeric flap, showing the descending branch of the lateral circumflex artery (yellow arrow), the musculocutaneous perforator (green arrow) to the ALT flap (black arrow), and the musculocutaneous perforator (white arrow) to the VL muscle (red arrow); (c) Transposition of the chimeric ALT-VL flap to the recipient site through a subcutaneous tunnel (grey arrow), where VL muscle filled the deep cavity (red arrow), and the ALT flap covered the superficial defect (black arrow); (d) Postoperative result

Therefore, in agreement with the patient, we decided to proceed with the reconstruction of the affected areas in two separate operations. The first operation involved the reconstruction of the left trochanteric area with a pedicled chimeric ALT-VL flap so that the muscle can fill the cavity of the resulting defect from the debridement of the trochanteric bursa and the fasciocutaneous part of the flap to cover the skin and soft tissue defect. The flap size was approximately 13 cm x 9 cm and was centered over the selected perforators, while the marked size of the defect was 10 cm x 8 cm (Figure 1). Initially, radical debridement of the radionecrotic and surrounding devitalized tissue was performed (Figure 2a). At the trochanteric area, the debridement reached the affected articular bursa with complete resection of the fistula. Wound swabs and tissue cultures were not taken since preoperative and intraoperative findings were negative for microbial colonization. Then, the flap was harvested, starting with a medial incision extended deep to the subfascial plane until the perforators were located (Figure 2a). We selected the most reliable distal musculocutaneous perforator to obtain the longest pedicle possible and to maximize the arc of the flap rotation. Therefore, we followed the route of the perforator towards the septum between the rectus femoris and vastus lateralis muscles to reach the descending branch of the lateral circumflex artery, which is the main vascular pedicle (Figure 2b). The lateral incision of the flap was then made superficial to the fascia lata, and the dissection proceeded medially until the elevation of the flap. In the case of the chimeric ALT-VL flap, the VL muscle was harvested on one perforator and then separated from the skin paddle (Figure 2b). Afterward, the pedicled ALT-VL flap was transposed into the trochanteric area via a subcutaneous tunnel. The VL muscle was used to fill the deep cavity, while the ALT flap was sutured superficially to cover the trochanteric defect (Figure 2c). Finally, the donor and recipient sites were closed primarily (Figure 2d).

**Figure 3:**
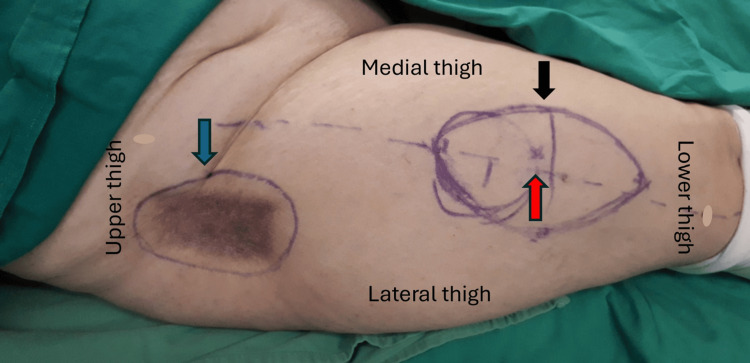
Preoperative markings at the right side showing the anterolateral thigh flap (black arrow), the detection of the most distal perforator (red arrow), and the area of radionecrosis at the trochanteric region (blue arrow). The flap size was approximately 15 cm x 10 cm, and the marked size of the defect was 12 cm x 10 cm.

**Figure 4:**
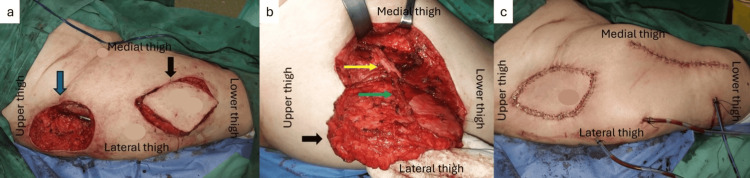
Intraoperative details of the reconstruction of the right trochanteric area (a) Harvesting of the fasciocutaneous anterolateral thigh (ALT) flap (black arrow), and the resulting defect at the trochanteric area after radical debridement (blue arrow); (b) Dissection of the fasciocutaneous ALT flap (black arrow), showing the descending branch of the lateral circumflex artery (yellow arrow) and the musculocutaneous perforator to the ALT flap (green arrow); (c) Postoperative result

After 10 months, we proceeded with the reconstruction of the right trochanteric area using the same surgical technique. The flap size was approximately 15 cm x 10 cm, and the marked size of the defect was 12 cm x 10 cm (Figure 3). At this site, we harvested a pedicled fasciocutaneous ALT flap based on one distal musculocutaneous perforator (Figures 4a, 4b). The trochanteric defect and the donor site were closed in the same way as on the left side (Figure 4c).

The total operating time was approximately five hours on the right side and six hours on the left side. Although the blood loss was minimal, the patient received a single unit of blood transfusion at each operation. During the early postoperative course, no complications occurred, and the patient was discharged after seven days, with instructions to remain in a supine or prone position as well as to avoid lateral decubitus and hip flexion for four weeks. The drains were removed after seven days, and antibiotic therapy was continued for two weeks.

**Figure 5:**
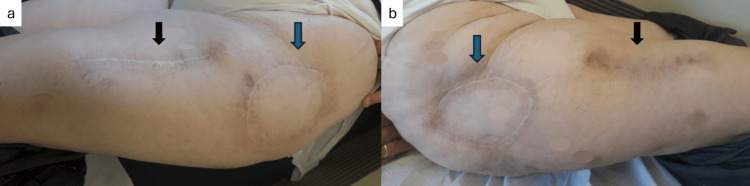
Postoperative view after 24 months of follow-up: (a) at the left side, and (b) at the right side with good esthetic and functional outcome both in the trochanteric (blue arrows) and donor areas (black arrows), without evidence of recurrence

**Figure 6:**
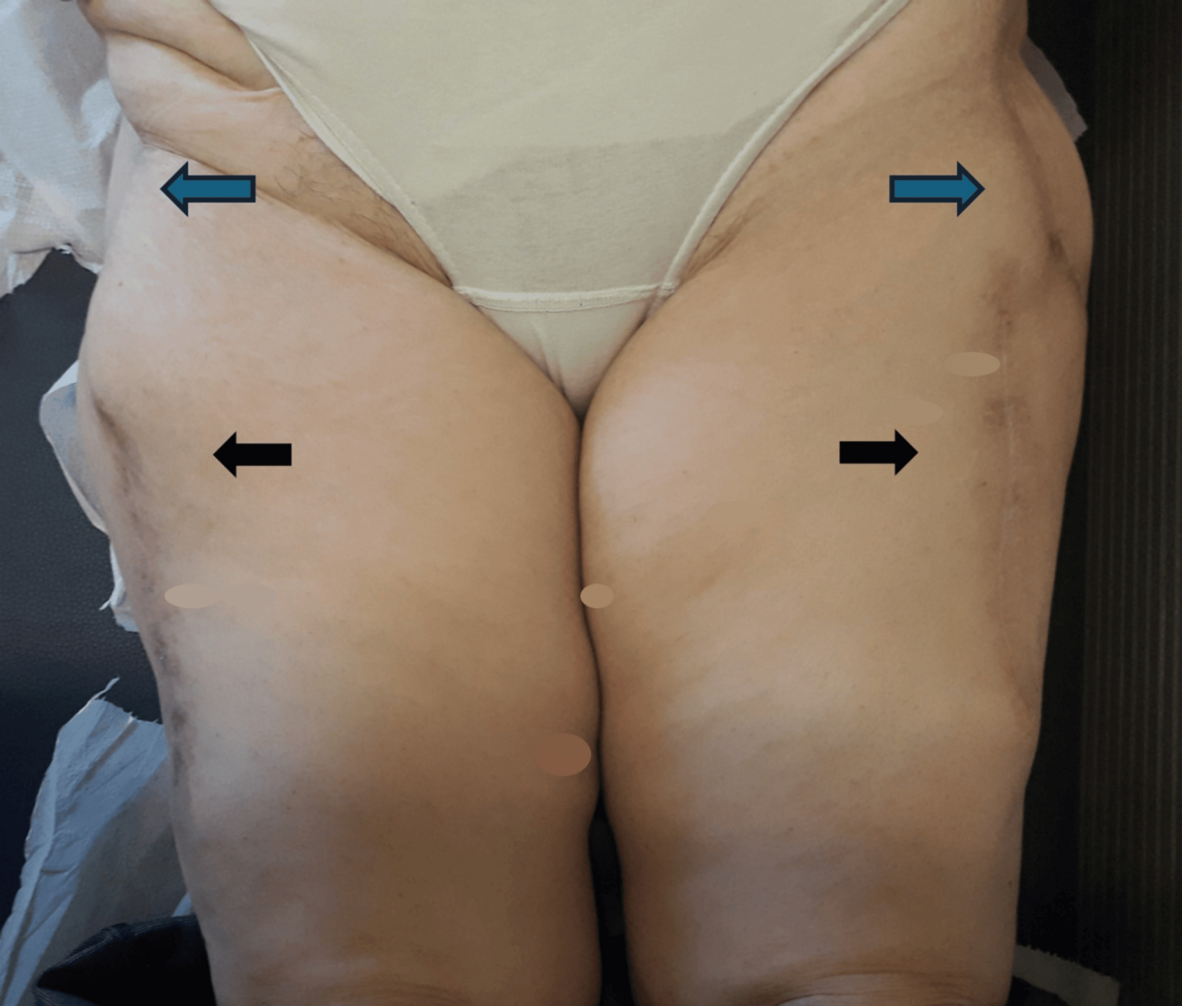
Postoperative view after 24 months of follow-up showing good healing and functional results at the trochanteric (blue arrows) and donor areas (black arrows).

Throughout the postoperative follow-up, we observed a significant reduction in flap edema, with the formation of a stable scar both in the donor and recipient areas, and there was no need for additional interventions. The flaps survived completely with adequate trochanteric coverage, offering an excellent esthetic and functional result. After 24 months of follow-up, the trochanteric areas were healed uneventfully without signs of recurrence (Figures 5, 6). The patient no longer reported pain, with improvement in hip movements and normal ambulation. Particularly, the hip flexion was approximately 100° with knees in flexion, while the hip extension was approximately 10° and hip abduction was approximately 40°. 

## Discussion

Reconstruction of the trochanteric area can be challenging since large and deep defects are usually present in patients with pressure sores and post-infective hip replacement surgery [4,5]. However, pelvic radiotherapy can induce late complications, mainly affecting the bone structures and presenting as osteoradionecrosis of the sacral area and hip [6,7]. To the best of our knowledge, this is a rare case of late bilateral skin and soft tissue radionecrosis of the trochanteric area without bone injury, successfully reconstructed with a combination of fasciocutaneous ALT flap and chimeric ALT-VL flap. In our case, the patient developed skin and soft tissue necrosis 30 years after radiotherapy. Similarly, Kim et al. described a case of infected gluteal osteoradionecrosis with a mean interval between operation and radiotherapy of 28.3 ± 8.3 years [8], while Hoang et al. reported a prospective study of radiation-induced ulcers with a median interval between radiotherapy and ulcer development of 7.5 years (range: one to 31 years) [9]. The pedicled ALT flap is a versatile flap for the reconstruction of regional defects, offering a long and reliable vascular pedicle, a wide arc of rotation, a large skin and bulky soft tissue area, the possibility to combine different tissues, and minimal donor site morbidity [4,10].

Initially, we performed an extensive debridement of the necrotic tissue and additionally a bursectomy on the left side to effectively treat the presence of the fistula. Interestingly, we didn’t experience any bone or soft tissue infection, which was also confirmed preoperatively with the MRI and laboratory blood tests. Therefore, we planned for one-stage surgical reconstruction for each side, first with a pedicled chimeric ALT-VL flap at the left trochanteric region to fill the deep cavity with the VL muscle, and 10 months later with a pedicled fasciocutaneous ALT flap at the right trochanteric region. In cases of radiation-induced wound infection, a two-stage surgical approach seems to be the best option, including initially serial radical debridement and eventually the use of negative-pressure wound therapy, and after resolution of the infection, the reconstruction of the defect with a muscle-based flap [8,11]. 

Technically, we propose to use the most distal musculocutaneous perforator to obtain a long vascular pedicle and facilitate the rotation of the flap, avoiding any tension during the closure of the trochanteric defect. Additionally, we spared the ascending branch of the lateral circumflex femoris artery to preserve the tensor fascia lata (TFL) flap for secondary reconstruction in case of recurrence. Moreover, we transferred the flaps through a subcutaneous tunnel, which is more suitable for elderly patients [12].

Although the TFL flap remains the most used flap for trochanteric reconstruction [13], we demonstrated that the ALT flap could be a safe and reliable alternative technique. Interestingly, the TFL flap presents several disadvantages, including defect coverage with the most distal and poorly vascularized portion, which can result in wound dehiscence and unstable scars, dog-ear deformity, poor cosmetic results at the donor site due to the need for skin grafts, and higher recurrence rates compared to the ALT flap [4,14,15]. Moreover, the pedicled ALT flap is easier to harvest, with significantly less operative time and lower morbidity compared to free flaps [11,16].

In our case, we transferred the VL muscle as a pedicled chimeric ALT-VL flap to achieve better rotation and flap setting and therefore to obliterate the dead space at the level of the bursectomy at the left trochanteric region. The VL muscle offers a bulky and well-vascularized tissue, able to protect the underlying bone structures, limiting the formation of seroma and hematoma, and preventing infection and therefore wound recurrence [5,17]. Particularly, radiation-induced ulcers and osteoradionecrosis present a high complication rate, and the use of myocutaneous flaps can provide adequate tissue to replace the lack of volume and prevent recurrence [8]. The VL muscle was covered by the fasciocutaneous ALT flap, and it wasn’t necessary to cover it with a skin graft [18], achieving a good esthetic result without affecting the patient’s posture and ambulation.

## Conclusions

Radical debridement and reconstruction with a well-vascularized pedicled or free flap are generally required for the reconstruction of long-lasting radionecrotic lesions. We recommend using a muscle component as part of a myocutaneous or chimeric flap in cases of deep and infected wounds to prevent recurrence and achieve good functional and esthetic outcomes. Our surgical approach aims to contribute valuable evidence to the limited scientific data on the treatment of post-radiotherapy tissue injuries, highlighting the importance of using the pedicled fasciocutaneous ALT and chimeric ALT-VL flaps for trochanteric skin and soft tissue radionecrosis reconstruction.
